# Cellular Inhibitor of Apoptosis (cIAP)-Mediated Ubiquitination of Phosphofurin Acidic Cluster Sorting Protein 2 (PACS-2) Negatively Regulates Tumor Necrosis Factor-Related Apoptosis-Inducing Ligand (TRAIL) Cytotoxicity

**DOI:** 10.1371/journal.pone.0092124

**Published:** 2014-03-14

**Authors:** Maria Eugenia Guicciardi, Nathan W. Werneburg, Steven F. Bronk, Adrian Franke, Hideo Yagita, Gary Thomas, Gregory J. Gores

**Affiliations:** 1 Division of Gastroenterology and Hepatology, College of Medicine, Mayo Clinic, Rochester, Minnesota, United States of America; 2 Department of Immunology, School of Medicine, Juntendo University, Tokyo, Japan; 3 Department of Microbiology and Molecular Genetics, University of Pittsburgh, Pittsburgh, Pennsylvania, United States of America; The University of Texas MD Anderson Cancer Center, United States of America

## Abstract

Lysosomal membrane permeabilization is an essential step in TRAIL-induced apoptosis of liver cancer cell lines. TRAIL-induced lysosomal membrane permeabilization is mediated by the multifunctional sorting protein PACS-2 and repressed by the E3 ligases cIAP-1 and cIAP-2. Despite the opposing roles for PACS-2 and cIAPs in TRAIL-induced apoptosis, an interaction between these proteins has yet to be examined. Herein, we report that cIAP-1 and cIAP-2 confer TRAIL resistance to hepatobiliary cancer cell lines by reducing PACS-2 levels. Under basal conditions, PACS-2 underwent K48-linked poly-ubiquitination, resulting in PACS-2 proteasomal degradation. Biochemical assays showed cIAP-1 and cIAP-2 interacted with PACS-2 *in vitro* and co-immunoprecipitation studies demonstrated that the two cIAPs bound PACS-2 *in vivo*. More importantly, both cIAP-1 and cIAP-2 directly mediated PACS-2 ubiquitination in a cell-free assay. Single *c-Iap-1* or *c-Iap-2* gene knock-outs in mouse hepatocytes did not lead to PACS-2 accumulation. However, deletion of both cIAP-1 and cIAP-2 reduced PACS-2 ubiquitination, which increased PACS-2 levels and sensitized HuH-7 cells to TRAIL-induced lysosomal membrane permeabilization and apoptosis. Correspondingly, deletion of cIAPs sensitized wild-type, but not PACS-2-deficient hepatocarcinoma cells or *Pacs-2^−/−^* mouse hepatocytes to TRAIL-induced apoptosis. Together, these data suggest cIAPs constitutively downregulate PACS-2 by polyubiquitination and proteasomal degradation, thereby restraining TRAIL-induced killing of liver cancer cells.

## Introduction

The inhibitor of apoptosis proteins (IAPs) are evolutionarily conserved and implicated in a variety of cellular processes, including repressing apoptosis in response to both extrinsic (death receptors-mediated) and intrinsic (cell stress-mediated) signaling pathways [1], [2]. IAP family members share structural homology in at least one baculovirus IAP repeat (BIR) domain, a stretch of ∼70 amino acids essential for protein-protein interactions. The mammalian IAPs, cellular IAP protein 1 and 2 (cIAP-1 and cIAP-2) and X chromosome-linked IAP (XIAP), contain three BIR domains in their amino-terminal regions. In addition, these IAPs bear a carboxyl-terminal RING (really interesting new gene) domain that confers E3 ubiquitin ligase activity, and a ubiquitin-associated (UBA) domain that enables binding of ubiquitin moieties [3]. Despite early observations that all three IAPs can directly bind caspases *in vitro*, only XIAP is actually a potent inhibitor of caspase activity *in vivo* [4], [5]. On the contrary, cIAP-1 and -2 exert their pro-survival effect mainly by means of their E3 ubiquitin ligase activity. They ubiquitinate specific substrates, either targeting the proteins for proteasomal degradation by catalyzing K48-linked polyubiquitination or modulating signaling pathways by catalyzing K63-linked polyubiquitination [3].

IAP proteins are frequently overexpressed in human malignancies and contribute to chemoresistance and poor prognosis [6]. These observations sparked the development of pharmacological inhibitors of IAPs for potential use in cancer therapy. Small molecules that mimic the IAP-binding motif of second mitochondrial activator of caspases (SMAC, also known as DIABLO), the endogenous IAP antagonist [7], [8], named SMAC mimetics, have recently been developed and are being tested in human clinical cancer trials. SMAC mimetics bind to the BIR2 and BIR3 domains of IAPs and inhibit IAPs via two distinct mechanisms: they can prevent binding of XIAP to active caspases [9] and they can induce autoubiquitination and rapid proteasomal degradation of cIAPs [9], [10], [11]. Pre-clinical studies have demonstrated the ability of different SMAC mimetics to either exert single agent cytotoxicity or sensitize tumor cells to pro-apoptotic stimuli [6], [12], [13], [14]. We recently reported that depletion of cIAP-1 by a SMAC mimetic or by genetic knock-down overcomes the resistance of hepatobiliary cancer cells to tumor necrosis factor-related apoptosis-inducing ligand (TRAIL) cytotoxicity, and that cIAP-1 degradation is required for efficient TRAIL-induced apoptosis [15]. In these cells, TRAIL treatment induces lysosomal membrane permeabilization (LMP) followed by release of cathepsin B and other lysosomal enzymes into the cytosol, which is a crucial step in potentiating mitochondrial dysfunction [16], [17]. Released cathepsin B appears to play a pivotal role in this apoptotic pathway, as cathepsin B-deficient mice are resistant to TRAIL-mediated liver injury [18]. We have also demonstrated that TRAIL-induced LMP is, in part, mediated by the Bcl-2 proteins Bim and Bax [17]. TRAIL stimulates JNK-mediated phosphorylation of Bim, which promotes the formation of a lysosomal signaling complex (named PIXosome) with Bax and phosphofurin acidic cluster sorting protein 2 (PACS-2) [19], a member of the PACS family of multifunctional sorting proteins regulating membrane traffic within secretory pathways and interorganellar communication [20]. This complex triggers LMP and promotes apoptosis, in accordance with a previous observation that PACS-2 is essential for TRAIL-induced hepatocyte apoptosis in a model of viral hepatitis [21].

The present study aimed to investigate whether cIAPs may prevent TRAIL-induced apoptosis by inhibiting the formation of the PIXosome. We provide evidence that, in hepatobiliary cancer cells, PACS-2 levels are regulated by cIAP-mediated polyubiquitination and consequent proteasomal degradation. Depletion of cIAP-1 and cIAP-2 results in PACS-2 accumulation, facilitating LMP during TRAIL-induced cell death. This study identifies PACS-2 as a key substrate for cIAPs and provides new insight into the mechanisms underlying the antiapoptotic effect of IAP proteins in TRAIL cytotoxic signaling.

## Experimental Procedures

### Reagents

Human recombinant TRAIL (375-TEC) was from R&D Systems (Minneapolis, MN). The proteasome inhibitor MG132 was from Calbiochem (La Jolla, CA). The SMAC mimetic JP1584 was from Joyant Pharmaceuticals (Dallas, TX). All other reagents were from Sigma-Aldrich (St. Louis, MO), unless otherwise specified.

### Antibodies

Rabbit polyclonal anti-PACS-2 (Ab193) was generated as previously described [22]. Mouse monoclonal anti-S-peptide was a generous gift from Dr. S. Kaufmann (Mayo Clinic, Rochester, MN). The other primary antibodies for immunoblot analysis were obtained from the following companies: rabbit polyclonal anti-SAPK-JNK (9252) and rabbit polyclonal anti-phospho SAPK-JNK (Thr183/Tyr185; 9251) from Cell Signaling (Beverly, MA); goat polyclonal anti-cIAP-1 (AF8181) from R&D Systems; rabbit monoclonal anti-cIAP-2 (clone E40; ab32059) from AbCam (Cambridge, MA); rat monoclonal anti-HA tag (clone 3F10; 1-867-423) from Roche Applied Science (Indianapolis, IN); rabbit monoclonal anti-TRAF2 (5525-1) from Epitomics (Burlingame, CA); mouse monoclonal anti-lysosomal associated membrane protein 1 (LAMP1) from BD Pharmingen (San Diego, CA); goat polyclonal anti-actin (sc-1615), mouse monoclonal anti-ubiquitin (clone P4D1; sc-8017), rabbit polyclonal anti-GST (sc-459), mouse monoclonal anti-His-probe (sc-8036); rabbit polyclonal anti-cathepsin B (sc-13985), goat polyclonal anti-cathepsin D (sc-6486) and goat polyclonal anti-cathepsin L (sc-6498) from Santa Cruz Biochemicals (Santa Cruz, CA); rabbit monoclonal anti-ubiquitin Lys63-specific (clone Apu3; 05-1308) and anti-ubiquitin Lys48-specific (clone Apu2; 05-1307) from Millipore (Billerica, MA). All primary antibodies were used at a concentration of 1 µg/ml, except actin and total ubiquitin (0.25 µg/ml), and His-probe, GST and cIAP-1 (0.5 µg/ml).

### Cell Lines and Primary Mouse Hepatocytes

Hepatocellular carcinoma cell line HuH-7 [23] and derived clones (HA-PACS-2-HuH-7, S-PACS-2-HuH-7, shPACS-2-HuH-7) [19], cholangiocarcinoma cell lines KMCH-1 and Mz-ChA-1 (both a kind gift from Dr. Nicholas LaRusso, Mayo Clinic, and obtained from Dr. M. Kojiro, Kurume University School of Medicine, Kurume, Japan and Dr. A. Knuth, Mainz University, Mainz, Germany, respectively) [24], [25], MEFs and freshly isolated primary mouse hepatocytes were all maintained in Dulbecco's modified Eagle's medium (DMEM) supplemented with 10% fetal bovine serum (FBS), 100 µg/ml streptomycin and 100 units/ml penicillin. TRAF2 knockout (*Traf2^−/−^* KO) MEFs, cIAP-1/cIAP-2 double knockout (*cIap-1/cIap-2^−/−^* DKO) MEFs and respective matching-background wild-type MEFs were a generous gift from Dr. J. Silke (La Trobe University, Victoria, Australia) and were generated as previously described [26]. Primary mouse hepatocytes were isolated from C57BL/6 wild-type mice (The Jackson Laboratory, Bar Harbor, ME) or mice genetically deficient in cIAP-1 (*cIap-1* ^−/−^) [27] or cIAP-2 (*cIap-2* ^−/−^) [28], both obtained from Dr. J. Ashwell (NIH, Bethesda, MD), or PACS-2 (*Pacs-2* ^−/−^) [21]. Mouse hepatocytes were isolated by collagenase perfusion, purified by Percoll (Sigma) gradient centrifugation, and plated as primary cultures.

### Plasmids and Transient Transfection

The plasmids encoding N-terminally HA-tagged human PACS-2 (pcDNA3.1-HA-PACS-2) have been previously described [21]. The plasmid encoding N-terminally S-peptide/streptavidin-binding peptide-double tagged PACS-2 (pSPN+SBP-PACS-2) was constructed by inserting PCR-amplified DNA containing nucleotides 176–2845 of the human PACS-2 open reading frame (NCBI Reference Sequence: NM_015197) into the BamHI and EcoRI sites of pSPN+SBP (a kind gift of Dr. S. Kaufmann, Mayo Clinic) [29]. The insert was amplified with Platinum *Taq* DNA polymerase high fidelity (Invitrogen, Carlsbad, CA) using pcDNA3.1-HA-PACS-2 as template and the following primers: Fw: 5′- AAT GGA TCC TAT GGC CGA GCG AGG C -3′; Rv: 5′- AAT GAA TTC TAG AAG GTG GCC TT -3′, which generated new BamHI and EcoRI sites. pEBB-HA-cIAP1H_588_A (RING mutant) was generated as previously described [15]. The plasmids were prepared for transfection using a plasmid midiprep kit (Bio-Rad, Hercules, CA, USA), and were subjected to automated sequencing to verify the presence of the described mutations and the absence of additional unwanted mutations. Cells growing in antibiotic-free DMEM+10% FBS were transfected using FuGENE HD transfection reagent (Roche Applied Science) with HD/DNA ratio 5∶2, following the manufacturer's instructions. Cells were analyzed 48 hours after transfection.

### Generation of Stable Transfectants

HuH-7 cells stably expressing HA-PACS-2 or shRNA against PACS-2 were generated as previously described [19]. To obtain clones stably expressing S-peptide/SBP-double tagged PACS-2, HuH-7 cells were transfected with 1 µg/ml pSPN/SBP-PACS-2 using Lipofectamine 2000 (Invitrogen) in OptiMEM I (Gibco-Invitrogen, Carlsbad, CA). Forty-eight hours after transfection, fresh DMEM containing 1 µg/ml neomycin was added. Surviving clones were selected and individually cultured. Expression of S-peptide/SBP-double tagged PACS-2 in the clones was assessed by immunoblot analysis for S-peptide

### Immunoprecipitation and Pull-Down Assays

Cells were treated with MG132 (5 µM) for 4 hours, then washed in ice-cold PBS and solubilized in lysis buffer (50 mM HEPES [pH 7.2], 120 mM NaCl, 1 mM EDTA, 0.1% NP-40, 10% (w/v) glycerol, protease inhibitor cocktail) for 30 min on ice. When samples were analyzed for ubiquitinated PACS-2, MG132 (20 µM) and N-ethylmalemide (NEM, 10 mM) were also added to the lysis buffer. After centrifugation at 13,000×*g* for 15 min, the supernatants were recovered and the protein concentration was determined using the Bradford reagent (Sigma-Aldrich). In pull-down experiments, aliquots containing 1–1.5 mg of protein were incubated with EZview Red anti-HA affinity gel (Sigma-Aldrich) or S protein agarose (Novagen/EMD Millipore) overnight at 4°C under rotary agitation. In immunoprecipitation experiments, aliquots containing 1.5 mg of protein were incubated with 10 µg of anti-ubiquitin Lys63-specific or anti-ubiquitin Lys48-specific antibodies, or 5 µg of anti-cIAP-2 antisera for 2 hours at 4°C, then incubated overnight with protein A agarose beads (Millipore) at 4°C under rotary agitation. Pelleted proteins were solubilized in SDS sample buffer, boiled for 5 min, clarified by centrifugation, and subjected to SDS-PAGE and immunoblot analysis.

### Immunoblot analysis

Whole-cell lysates were obtained as previously described [30]. Protein concentration was determined using the Bradford reagent (Sigma-Aldrich). Aliquots containing 50 µg of protein were resolved by SDS-PAGE, transferred to nitrocellulose membrane and blotted with primary antibodies overnight at 4°C. HRP-conjugated secondary antibodies (Santa Cruz) were incubated at a dilution of 1∶3000 for 1 hour at room temperature. Bound antibodies were visualized using enhanced chemiluminescence reagents (ECL; GE Healthcare, Buckinghamshire, UK).

### Generation of recombinant proteins

Plasmids expressing GST, GST-PACS-2FBR_38–202_ corresponding to residues 38–202 (cargo/adaptor-binding region-FBR) [31] and pET15b-FBR2 plasmid expressing His_6_-PACS-2FBR_38–217_ [22] were induced in BL21-A1 *E. coli* (Invitrogen) with 1 mM isopropyl-β-D-thiogalactoside (Calbiochem, Gibbstown, NJ) and 0.2% L-arabinose (Sigma Aldrich) for 4 hours at 37°C. Bacterial pellets were resuspended in PBS (pH 7.4) containing 1 mM PMSF and 1 mM DTT, subjected to sonication, and incubated on ice for 30 min in the presence of 1% Triton X-100. Soluble material was recovered by centrifugation at 13,000×*g* for 20 min at 4°C and subsequently purified using GSTrap FF affinity columns (GE Healthcare, Piscataway, NJ) or HisPur Ni-NTA Spin Columns (Thermo Scientific/Pierce Biotechnology, Rockford, IL) following the manufacturer's instructions.

### *In Vitro* Binding Assay

Aliquots of recombinant GST-PACS-2 FBR_38–202_ or GST (∼200 ng) were incubated for 30 min at 4°C in 20 µl of assay buffer (20 mM Tris, pH 7.9; 150 mM NaCl; 0.1 mM EDTA, 0.1% NP-40) with recombinant cIAP-1 and/or cIAP-2 (500 ng; R&D Systems), in the presence or absence of recombinant His-TRAF2 (500 ng; Sigma Aldrich). At the end of the incubation, the volume of each sample was brought to 400 µl with assay buffer and GST-containing proteins were affinity-purified by adding 40 µl of GST-agarose beads (BioWorld, Dublin, OH) for 30 min at 4°C. The agarose beads were recovered after centrifugation at 3,200×*g* for 2 min, washed once in assay buffer and four times in PBS, and the bound complexes were eluted by adding equal volumes of electrophoresis sample buffer, boiling for 5 min and centrifuging at 8,000×*g* for 1 min to pellet the beads. The supernatants containing the eluted proteins were analyzed by immunoblot.

### *In Vitro* Ubiquitination Assay

Aliquots of recombinant His-PACS-2FBR_38–217_ (∼1 µg) were incubated for 1 hour at 37°C in 50 µl of assay buffer (30 mM HEPES, pH 7.5; 2 mM DTT; 10 µM ZnCl_2_; 5 mM MgCl_2_; 2 mM ATP) containing 250 ng of recombinant cIAP-1 or cIAP-2 (E3), 0.5 µg E1 (Ube1), and 1 µg E2 (UbcH5a), in the presence of 10 µg ubiquitin (all reagents from Boston Biochem, Cambridge, MA). At the end of the incubation, after addition of electrophoresis sample buffer, the samples were boiled for 5 min, centrifuged at 10,000×*g* for 1 min and analyzed by immunoblot.

### PACS-2 Translocation to Lysosomes and Assessment of Lysosomal Membrane Permeabilization (LMP)

Lysosomal fractions from cultured cells were obtained using a commercially available Lysosome Isolation Kit (Sigma-Aldrich) and analyzed by immunoblot.

LMP was assessed by immunoblot analysis for lysosomal enzymes (cathepsin B, cathepsin D and cathepsin L) in the cytosolic fractions, as well as by cathepsin B immunofluorescence in fixed cells. Cytosolic fractions were obtained by a selective digitonin permeabilization technique validated by Garrison and coworkers [32] with slight modifications. Briefly, cells were washed and collected in PBS containing a protease inhibitor cocktail (Roche), centrifuged at 1,200×*g* for 5 min and incubated on ice for 5 min in permeabilization buffer (20 mM HEPES, pH 7.2, 100 mM KCl, 5 mM MgCl_2_, 1 mM EDTA, 1 mM EGTA, 250 mM sucrose, protease inhibitor cocktail and 0.01% digitonin). This concentration of digitonin was chosen after validation studies demonstrated its ability to selectively permeabilize the plasma membrane, but not the lysosomal membrane (data not shown). Samples were subsequently centrifuged at 15,000×*g* at 4°C for 10 min, and the supernatants were collected and analyzed by immunoblot.

Cathepsin B immunofluorescence was performed as previously described [19], using a mouse monoclonal anti-cathepsin B antibody (clone CA10, #1M27L, Calbiochem/EMD Millipore) at dilution 1∶500. Cells were mounted using the ProLong Gold antifade reagent with DAPI (Molecular Probes), imaged by confocal microscopy (Zeiss LSM 510, Carl Zeiss Micro-Imaging Inc., Thornwood, NJ) with λ_em_ = 488 nm and λ_ex_ = 507 nm, and scored for their punctate versus diffuse fluorescence.

### Quantification of Apoptosis

Apoptosis was quantified by fluorescence microscopy after staining with 4′,6-diamidino-2-phenylindole dihydrochloride (DAPI, Sigma) as previously described [15].

### Animal Studies

All animal studies were performed in accord with and approved by the Mayo Clinic Institutional Animal Care and Use Committee. Three to four month-old C57BL/6 wild-type and *Pacs-2* ^−/−^ mice were given the mouse DR5-specific monoclonal antibody MD5-1 (300 µg/mouse) [33] intraperitoneally once on day 1 and once on day 4 of the study. Gender- and age-matched, untreated wild-type and *Pacs-2* ^−/−^ mice were used as controls. The mice were euthanized 18 days after the first injection and livers and blood were removed for analysis. Serum transaminase (alanine aminotransferase, ALT; aspartate aminotransferase, AST) activity was measured using standardized and automated procedures of the diagnostic laboratory at the Mayo Clinic. Tissue samples were cryopreserved in Tissue Tek OTC compound (Takeda, Deerfield, IL) immediately after removal.

### TUNEL Assay

Liver apoptotic cells were quantitated by the terminal deoxynucleotidyl transferase-mediated dUTP nick-end labeling (TUNEL) assay. Cryopreserved liver tissue sections were cut at 5 µm on a cryomicrotome (Leica, Buffalo Grove, IL) and air-dried. TUNEL assay was performed using the In Situ Cell Death Detection Kit with Fluorescein (Roche Diagnostics, Indianapolis, IN) following the manufacturer's protocol. TUNEL-positive cells were counted using an inverted laser scanning confocal microscope (Zeiss LSM 780) with λ_em_ = 480 nm and λ_ex_ = 525 nm. A total of five high-power fields (HPF) were analyzed for each animal.

### Statistical analysis

All data represent at least three independent experiments and are expressed as means ± standard errors (SE), unless otherwise indicated. Differences between groups were analized using one-way analysis of variance (ANOVA) for multiple groups; individual group means were compared with Student's unpaired *t* test. *P* values<0.05 were considered statistically significant.

## Results

### Posttranslational regulation of PACS-2 by proteasomal degradation

**Figure 1:**
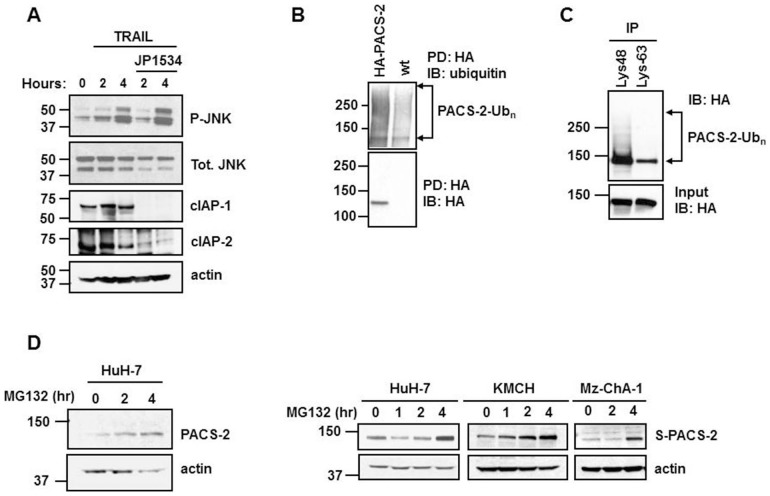
PACS-2 levels are regulated by K48-linked polyubiquitination and proteasomal degradation. (A) HuH-7 cells were treated with TRAIL (20 ng/ml) in the presence or absence of the SMAC mimetic JP1584 (500 nM) for up to 4 hours. Total cell lysates were analyzed by immunoblot with antisera against total and phospho-JNK (T183/Y185), cIAP-1, cIAP-2 and actin (loading control). (B) HuH-7 cells stably over-expressing HA-PACS-2 or wild-type HuH-7 cells were lysed and subjected to HA pull-down using HA agarose beads. The samples were analyzed by immunoblot with antisera against total ubiquitin. (C) HuH-7 cells over-expressing HA-PACS-2 were lysed and subjected to immunoprecipitation using antibodies against Lys48-linked or Lys63-linked poly-ubiquitin, then analyzed by immunoblot for HA. Total cell lysates show equal expression of HA-PACS-2 in the samples. (D) HuH-7 cells (left panel) or HuH-7, KMCH and Mz-ChA-1 cells transiently transfected with S-peptide/SBP-double tagged PACS-2 (right panel) were treated with MG132 (10 µM) for the indicated times. PACS-2 cellular levels were analyzed by immunoblot in total cell lysates.

The determination that JNK phosphorylation of Bim Ser_69_ is required for PIXosome assembly and LMP, together with the ability of cIAPs to regulate death receptor-induced JNK activation and apoptosis in liver cancer cells [15] [19] [34], led us to ask if cIAP inhibitors can block TRAIL-induced LMP by preventing Bim phosphorylation. Therefore, we examined TRAIL-mediated JNK activation in hepatocellular carcinoma HuH-7 cells in the presence or absence of the SMAC mimetic JP1584, which induces rapid degradation of cIAP-1 and cIAP-2. We found that JP1584 had no measurable effect on TRAIL-induced JNK activation, suggesting SMAC mimetics block TRAIL action downstream of Bim phosphorylation in this model (Fig. 1A).

We then tested the possibility that cIAPs regulate PACS-2. Initially, we asked whether PACS-2 may be ubiquitinated. Accordingly, HuH-7 cells stably expressing HA-tagged PACS-2 [19] were lysed and PACS-2 ubiquitination status was examined after HA immunoprecipitation and immunoblot analysis with an ubiquitin specific antibody. High molecular weight species visualized as a “smear” on the blot suggested PACS-2 was constitutively polyubiquitinated in these cells (Fig. 1B). To determine the ubiquitin linkage of the polyubiquitin chains on PACS-2, HA-PACS-2 cell lysates were subjected to immunoprecipition using anti-ubiquitin Lys48-specific or Lys63-specific antibodies, then subsequently immunoblotted using anti-HA antiserum. PACS-2 polyubiquitination was detected only in the pull-down with anti-ubiquitin Lys48-specific but not Lys63-specific antibody (Fig. 1C), suggesting the polyubiquitin chains on PACS-2 were linked primarily through lysine 48 of ubiquitin. As Lys48-linked polyubiquitination typically targets a protein for degradation via the proteasomal pathway, we postulated that inhibition of the proteasome would lead to cellular accumulation of PACS-2. Indeed, HuH-7 cells treated with MG132 displayed a time-dependent increase of both endogenous PACS-2 and stably expressed, exogenous S-peptide/SBP-double tagged PACS-2 (Fig. 1D). These results were not cell line specific, as virtually identical observations were made in the cholangiocarcinoma cell lines KMCH and Mz-ChA-1 (Fig. 1D). Thus, these findings indicate that PACS-2 cellular levels are in part regulated by K48-linked polyubiquitination and proteasomal degradation in hepatobiliary cancer cells.

### cIAPs ubiquitinate PACS-2 in vivo and in vitro, and promote its degradation

**Figure 2:**
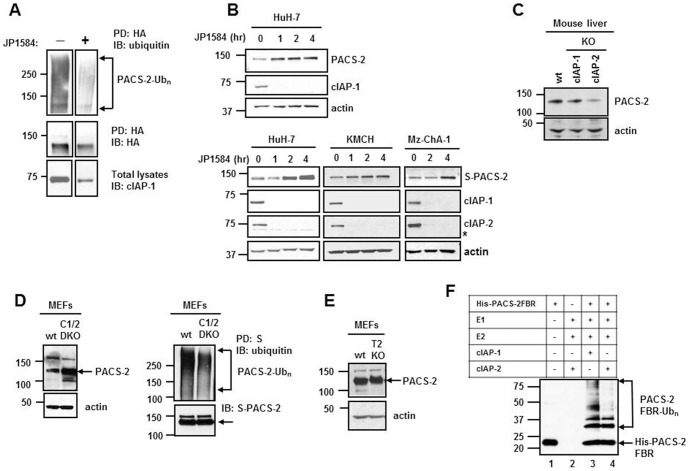
PACS-2 is ubiquitinated by cIAPs in vivo and in vitro. (A) HA-PACS-2-overexpressing HuH-7 cells were incubated with or without JP1584 (500 nM) for 1 hr, followed by HA pull-down and immunoblot analysis with antisera against total ubiquitin or HA. Total lysates prior HA pull-down were immunoblotted for cIAP-1. Images were cut and combined from the same film to remove adjacent unrelated lanes. (B) HuH-7 cells ( upper panel) and HuH-7, KMCH and Mz-ChA-1 cells transiently transfected with S-peptide/SBP-double tagged PACS-2 ( lower panel) were treated with JP1584 for the indicated times. PACS-2, cIAP-1 and cIAP-2 levels were analyzed by immunoblot. The asterisk (*) indicates a non-specific band. (C) Total liver lysates from wild-type, *cIap1* ^−/−^ or *cIap2* ^−/−^ mice and (D) *cIap1* ^−/−^ *cIap2* ^−/−^ DKO (c1/2 DKO) MEFs and matching background wild-type MEFs were analyzed by immunoblot for PACS-2 expression (left panel). *cIap1* ^−/−^ *cIap2* ^−/−^ DKO MEFs and wild-type MEFs were transiently transfected with S-peptide/SBP-double tagged PACS-2, subjected to S-peptide pull-down and analyzed by immunoblot with antisera against total ubiquitin or S-peptide (right panel). (E) *Traf2* ^−/−^ (T2 KO) and matching background wild-type MEFs were analyzed by immunoblot for PACS-2 expression. Actin was used as loading control. (F) An *in vitro* ubiquitination assay was performed by incubating His-PACS-2FBR_38–202_ with recombinant E1, UbcH5a (E2) and cIAP-1 or cIAP-2 (E3), in buffer containing wild-type ubiquitin and ATP, for 1 hour at 37°C. PACS-2FBR_38–202_ ubiquitination was detected by immunoblot analysis using antisera against the His-tag.

Given the role of degradative ubiquitination in PACS-2 regulation, we next sought to ascertain if cIAPs could act as E3 ubiquitin ligases for PACS-2. HA-PACS-2 HuH-7 cells were incubated in the presence or absence of JP1584 for 1 hour, subjected to HA immunoprecipitation and analyzed by immunoblot for PACS-2 ubiquitination (Fig. 2A). HA-PACS-2 polyubiquitination was markedly reduced in cells treated with the SMAC mimetic, suggesting that IAPs are critical E3 ligases responsible for PACS-2 ubiquitination (Fig. 2A). Treatment with JP1584 also resulted in time-dependent accumulation of both endogenous PACS-2 and S-peptide/SBP-double tagged PACS-2 in all cell lines (Fig. 2B, upper and lower panel, respectively), confirming that IAPs modulate PACS-2 degradation. To ascertain if PACS-2 ubiquitination was carried out specifically by cIAP-1 or cIAP-2, we examined PACS-2 protein levels in total liver lysates from *cIap1* ^−/−^ or *cIap2* ^−/−^ mice. Surprisingly, no accumulation of PACS-2 was detected in either sample compared to the wild-type liver lysate, suggesting depletion of both cIAP-1 and cIAP-2 may be necessary to reduce PACS-2 degradation (Fig. 2C). Given the unavailability of liver tissue from *cIap1* ^−/−^ *cIap2* ^−/−^ DKO mice as these mice die during embryonic development, we assessed the levels of PACS-2 in *cIap1* ^−/−^ *cIap2* ^−/−^ DKO MEFs and compared them with wild-type MEFs. Endogenous PACS-2 was indeed more abundant in unstimulated *cIap1* ^−/−^ *cIap2* ^−/−^ DKO MEFs compared to wild-type MEFs (Fig. 2D). Moreover, ubiquitination of exogenous PACS-2 was reduced in the *cIap1* ^−/−^ *cIap2* ^−/−^ DKO MEFs (Fig. 2D). Therefore, it appears that cIAP-1 and cIAP-2 act coordinately and redundantly to ubiquitinate PACS-2. TRAF2 binds to cIAP-1 and cIAP-2, recruiting them to different signaling complexes [35], and has been shown to stabilize cIAP-1 [36]. In order to verify whether the cellular levels of PACS-2 were affected by the absence of TRAF2, PACS-2 was analyzed in wild-type and *Traf2* ^−/−^ MEFs. PACS-2 levels were comparable in both cell lines, suggesting TRAF2 is dispensable for cIAP regulation of PACS-2 (Fig. 2E). Finally, we tested the ability of cIAP-1 and cIAP-2 to directly ubiquitinate PACS-2 in a cell-free ubiquitination assay. Given the difficulties to obtain recombinant full-length PACS-2 from a bacterial system due to extensive bacterial degradation, we generated recombinant His-PACS-2FBR_38–217_ which contains residues 38–217 corresponding to the cargo/adaptor-binding region-FBR of PACS-2. Purified His-PACS-2FBR was incubated with recombinant cIAP-1 or cIAP-2 in the presence of recombinant E1 and E2 (UbcH5a) enzymes, in a reaction buffer containing ubiquitin and ATP. Incubation of His-PACS-2FBR with cIAP-1 or cIAP-2 resulted in PACS-2FBR polyubiquitination (Fig. 2F), indicating that PACS-2 is a direct substrate for both cIAP-1 and cIAP-2.

### cIAPs bind to PACS-2 in vivo and in vitro

**Figure 3:**
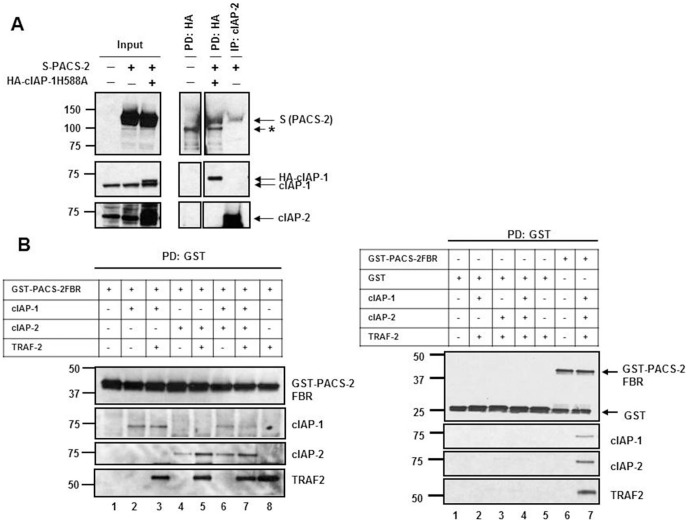
cIAP-1 and cIAP-2 bind to PACS-2 in vivo and in vitro. (A) HuH-7 cells untransfected or transiently transfected with S-peptide/SBP-double tagged PACS-2 ± HA-cIAP-1H588A expression plasmids were subjected to HA (cIAP-1) pull-down or cIAP-2 immunoprecipitation. Affinity-purified proteins and total cell lysates (input) were analyzed by immunoblot for S-peptide (PACS-2), cIAP-1 and cIAP-2. Images were cut and combined from the same film to remove adjacent unrelated lanes. The asterisk (*) indicates a non-specific band. (B) An *in vitro* binding assay was performed by incubating recombinant GST or GST-PACS-2FBR_38–202_ with recombinant cIAP-1 or cIAP-2, in the presence or absence of recombinant human His-TRAF2, for 30 min at 4°C. GST was subsequently pulled-down and the precipitates were analyzed by immunoblot with the indicated antibodies.

To confirm that IAPs and PACS-2 physically interact, cells were co-transfected with S-peptide/SBP-double tagged PACS-2 and HA-tagged cIAP-1 RING mutant, in which His_588_ had been mutated to Ala, abrogating the E3 ubiquitin ligase activity of cIAP-1. The use of the RING mutant was necessary in order to achieve higher expression of the exogenous HA-cIAP-1, as the wild-type protein undergoes rapid auto-ubiquitination and proteasomal degradation when overexpressed [36]. Pull-down of HA-cIAP-1 displayed an interaction between cIAP-1 and PACS-2 (Fig. 3A). Moreover, immunoprecipitation of endogenous cIAP-2 also demonstrated association of PACS-2 with cIAP-2 (Fig. 3A). To determine whether cIAPs bind directly to PACS-2 or may require another co-factor (i.e., TRAF2), we assessed the ability of PACS-2 to co-precipitate with cIAP-1 or cIAP-2 in a cell-free system using purified components. Purified recombinant GST-PACS-2FBR (Fig. 3B, left panel) or GST (used as control to assess nonspecific binding; Fig. 3B, right panel) were incubated with cIAP-1 or cIAP-2, in the presence or absence of recombinant His-TRAF2; GST-containing proteins were subsequently immunoprecipitated using GST-agarose beads and the resulting samples were subjected to immunoblot analysis. Despite the finding that TRAF2 does not seem to be required for cIAP-mediated downregulation of PACS-2 (Fig. 2E), cIAP-1, cIAP-2 and TRAF2 all bound GST-PACS-2-FBR *in vitro*, alone or in combination (Fig. 3B); likely, TRAF2 is associating with cIAP-1 and -2 in this cell-free system as has been previously demonstrated [37]. The binding of cIAP-1, cIAP-2 or TRAF2 did not occur in the GST region of GST-PACS-2-FBR, as GST alone did not associate with any of the proteins (Fig. 3B, right panel).

### PACS-2 accumulation facilitates TRAIL-induced LMP

**Figure 4:**
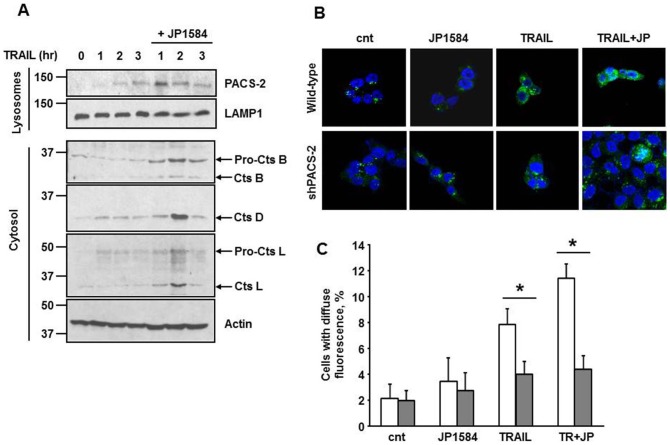
PACS-2 accumulation facilitates its translocation to the lysosomes. (A) HuH-7 cells were treated with TRAIL (10 ng/ml) ± JP1584 (500 nM). At the indicated times, lysosomal and cytosolic fractions were obtained and subjected to immunoblot analysis. LAMP1, an integral lysosomal membrane protein, and actin were used as loading control in the lysosomal and cytosolic fractions, respectively. (B) Wild-type and shPACS-2 HuH-7 cells were treated with TRAIL ± JP1584 for 3 hours and intracellular localization of cathepsin B was detected by immunofluorescence and imaged by confocal microscopy. (C) Quantification of the cells with permeabilized lysosomes was performed by scoring for punctate or diffuse appearance of cathepsin B and is expressed as percent of total number of cells per field. *p<0.02.

Because PACS-2 trafficking to lysosomes promotes TRAIL-mediated LMP [19], we speculated that cellular accumulation of PACS-2 may increase sensitivity to TRAIL-induced LMP. To test this possibility, lysosomal fractions were prepared from HuH-7 cells treated with TRAIL in the presence or absence of JP1584, and analyzed by immunoblot for PACS-2. PACS-2 was translocated to lysosomes more rapidly and efficiently when cells were incubated with TRAIL plus JP1584 compared to TRAIL alone (Fig. 4A). Notably, the increased translocation of PACS-2 to the lysosomes in the presence of the SMAC mimetic was coupled with an accelerated onset of LMP, as demonstrated by release of lysosomal cathepsin B, D and L into the cytosol (Fig. 4A). The membrane fractionation studies were corroborated by confocal microscopy. Parallel plates of Huh-7 WT and shPACS-2 cells were treated with TRAIL or JP1584 alone or in combination and the subcellular distribution of cathepsin B was monitored by cell imaging. In control cells, cathepsin B displayed a punctate staining pattern characteristic of lysosomes. Addition of JP1584 alone did not change cathepsin B localization. However, following treatment with TRAIL, the cathepsin B staining pattern became diffuse, consistent with lysosome permeabilization and release of cathepsin B into the cytoplasm (Fig. 4B and C). Addition of JP1584 to TRAIL further increased the amount of diffuse cytosolic staining, suggesting the two compounds cooperate to induce LMP. By contrast, HuH-7 cells with targeted shRNA knockdown of PACS-2 [19] were resistant to LMP mediated by TRAIL and JP1584, confirming that PACS-2 plays a key role in the induction of LMP (Fig. 4B and C).

### PACS-2 Deficiency Protects from TRAIL-induced Hepatocyte Apoptosis and Liver Injury and Prevents Sensitization by the SMAC Mimetic

**Figure 5:**
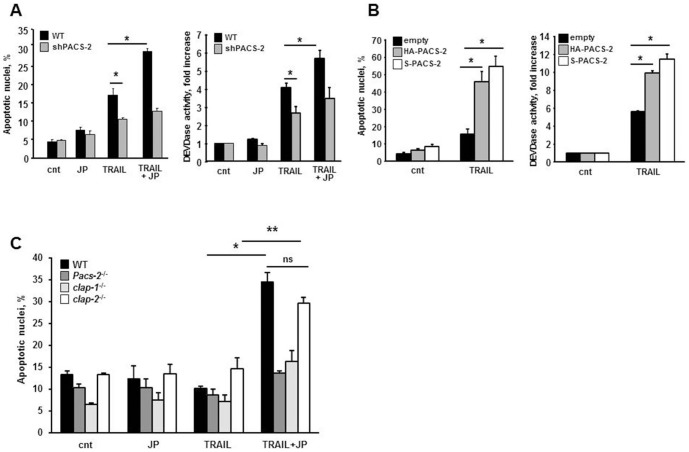
PACS-2 deficiency protects from TRAIL-induced apoptosis and prevent sensitizations by the SMAC mimetic. (A) Wild-type (WT) or shPACS-2 HuH-7 cells were treated with TRAIL (10 ng/ml) ± JP1584 (500 nM) for 6 hr. Apoptosis was assessed morphologically after DAPI staining (left panel) and by measuring caspase 3/7 activation (DEVDase activity; right panel). (B) HuH-7 cells were transiently transfected with plasmids encoding HA-PACS-2 or S-PACS-2 or with an empty plasmid (pcDNA3.1) as control for 48 hours, then treated with TRAIL (10 ng/ml) ± JP1584 (500 nM) for 6 hr. Apoptosis was assessed morphologically after DAPI staining (left panel) and by measuring caspase 3/7 activation (right panel). DEVDase activity is expressed as fold increase of relative fluorescent units over control value (untreated). *p<0.01. (C) Primary mouse hepatocytes from wild-type (WT), *Pacs-2* ^−/−^, *cIap1* ^−/−^ or *cIap2* ^−/−^ KO mice were treated with TRAIL (25 ng/ml) ± JP1584 for 8 hr. Apoptosis was assessed morphologically after DAPI staining. *p<0.001; **p<0.01, ns = non significant.

**Figure 6:**
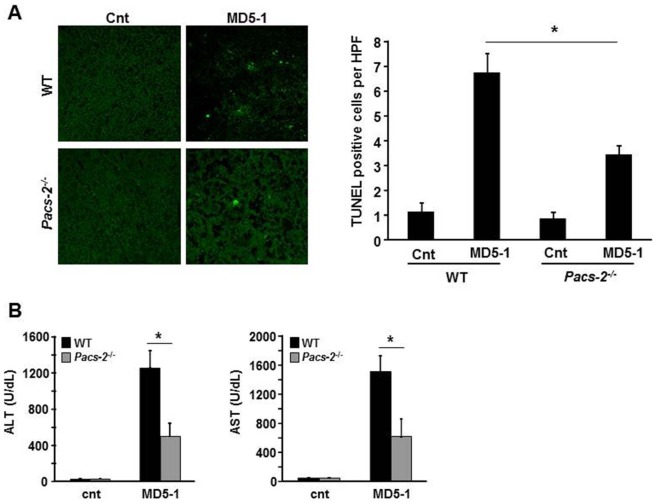
PACS-2 deficiency protects from anti DR5 antibody-induced liver injury. C57BL/6 wild-type (WT) and *Pacs-2^−/−^* mice (n = 5 per group) were injected with MD5-1 (300 µg/mouse) intraperitoneally on day 1 and day 4, then sacrificed at day 18. Gender- and age-matched untreated wild-type or *Pacs-2^−/−^* mice were used as controls (cnt). (A) Hepatocyte apoptosis in frozen liver tissue was analyzed by TUNEL assay. The number of TUNEL-positive cells was quantitated in 5 random microscopic high-power fields (HPF) per animal. (B) Serum ALT and AST levels were measured by standard techniques. *p<0.05.

To test whether the absence of PACS-2 also confers resistance to TRAIL-induced hepatocyte apoptosis, we first treated WT and shPACS-2 HuH-7 cells with TRAIL in the presence or absence of JP1584. Consistent with the observation that PACS-2 is necessary for LMP, shPACS-2 cells were also resistant to TRAIL-induced apoptosis and were not sensitized by the SMAC mimetic (Fig. 5A). Conversely, cells over-expressing PACS-2 displayed enhanced sensitivity to TRAIL-induced apoptosis (Fig. 5B). Next, we conducted a side-by-side analysis of primary hepatocytes isolated from WT, *cIap-1* ^−/−^, *cIap-2* ^−/−^, or *Pacs-2* ^−/−^ mice. Cultured primary hepatocytes do not undergo apoptosis following treatment with several forms of recombinant soluble TRAIL or TRAIL-receptor agonists [38]. Our previous studies indicated the same soluble TRAIL used in this study was unable to induce significant apoptosis in primary rat hepatocytes [39]. In agreement with these observations, WT mouse hepatocytes displayed no apoptosis in response to TRAIL or JP1584 alone. However, WT hepatocytes were sensitized to apoptosis when the two molecules were added in combination (Fig. 5C). By contrast, primary hepatocytes from *Pacs-2* ^−/−^ mice were resistant to TRAIL and JP1584 added singularly or together. Primary hepatocytes from *cIap1* ^−/−^ or *cIap2* ^−/−^ mice were also resistant to TRAIL-induced apoptosis, demonstrating that the absence of either protein is not sufficient to sensitize the cells to TRAIL. However, JP1584 sensitized *cIap-2* ^−/−^ hepatocytes to TRAIL, whereas the *cIap-1* ^−/−^ hepatocytes retained their resistance (Fig. 5C). Finally, we verified whether mice genetically deficient in PACS-2 were protected from TRAIL-induced hepatocyte apoptosis and liver damage *in vivo*. In an experimental model employing a mouse DR5-specific antibody (MD5-1) capable of inducing liver damage [40], *Pacs-2* ^−/−^ mice displayed significantly less apoptosis and liver damage compared to C57BL/6 wild-type mice as assessed by TUNEL assay and serum transaminase levels, respectively (Fig. 6A and B). Collectively, these data indicate that PACS-2 is required for TRAIL-induced LMP and cell death, and demonstrate that accumulation of PACS-2 in the absence of cIAPs facilitates its translocation to lysosomes and sensitizes cells to TRAIL killing.

## Discussion

The results of this study identify a novel mechanism by which cIAPs mediate resistance to TRAIL-induced apoptosis in liver cancer cells, namely, by regulating the formation of the PACS-2, Bim, Bax-containing signaling complex PIXosome on the lysosomes. The principal findings indicate that (i) PACS-2 is constitutively polyubiquitinated and degraded via the proteasome pathway in liver cells; (ii) cIAPs bind to and directly ubiquitinate PACS-2; (iii) pharmacological depletion (by SMAC mimetics) or genetic deletion of both cIAP-1 and cIAP-2 impairs PACS-2 ubiquitination and results in its intracellular accumulation; and (iv) PACS-2 accumulation facilitates its translocation to the lysosomes following TRAIL treatment, promoting TRAIL-induced LMP and apoptosis. Each of these observations is discussed in greater detail below.

We have reported that PACS-2 is required for TRAIL-induced apoptosis and that inhibition of IAPs, in particular cIAP-1, sensitizes liver cancer cells to TRAIL-induced apoptosis [15], [19]. Whether these molecules interact with and/or regulate each other, however, was not investigated in those studies. cIAPs possess E3 ubiquitin ligase activity and are known to modulate ubiquitin-dependent signaling events triggered by death receptor activation. Herein, we provide data suggesting PACS-2 is ubiquitinated via Lys48-linked poly-ubiquitin chains in untreated hepatobiliary cancer cells, which results in its proteasomal degradation. Consequently, PACS-2 cellular levels are repressed in these cells. Our findings suggest cIAPs function as E3 ligases for PACS-2 ubiquitination, as cellular depletion of cIAP-1 and cIAP-2 by a SMAC mimetic reduced PACS-2 ubiquitination and resulted in PACS-2 accumulation. Our previous studies pointed to a prominent role for cIAP-1 in TRAIL-resistance in liver cancer cells [15]; therefore, we anticipated that cIAP-1 would be mainly responsible for PACS-2 ubiquitination. Unexpectedly, both cIAP-1 and cIAP-2 ubiquitinate PACS-2 in a redundant manner, as the absence of either protein alone does not affect PACS-2 ubiquitination or protein stability. Only the simultaneous depletion of cIAP-1 and cIAP-2, as observed following the use of a SMAC mimetic or in cIAP-1^−/−^cIAP-2^−/−^ DKO MEFs, results in inefficient PACS-2 ubiquitination and accumulation within the cell. cIAP depletion has also been reported to indirectly enhance cFLIP_L_ degradation, thereby facilitating caspase 8 activation [41], a process which would also promote TRAIL pro-apoptotic signaling. Thus, it is likely that cIAPs exert their anti-apoptotic effect on multiple signaling pathways.

In agreement with the observation that PACS-2 is a substrate for cIAPs, immunoprecipitation studies identified direct binding between PACS-2 and cIAP-1 and cIAP-2 both *in vivo* and in cell-free systems. TRAF2 has been shown to stabilize cIAP-1 [36], and is required for cIAP-mediated degradation of NIK, as well as for SMAC mimetic-induced cIAP autoubiquitination and degradation [42], [43]. Interestingly, although TRAF2 co-precipitates with PACS-2 in cell-free systems, in the presence or absence of cIAP-1 and/or cIAP-2, it seems to be dispensable for cIAP ubiquitination and downregulation of PACS-2. Moreover, although TRAF2 was readily co-precipitated with cIAP-1, we were unable to identify TRAF2 in complex with PACS-2 in cell lysates (data not shown). These observations suggest that cIAP-1 and cIAP-2 might independently associate with PACS-2 and TRAF2 in different intracellular pools, and that TRAF2 is not recruited to the PACS-2∶cIAP-1∶cIAP-2 complex *in vivo*.

PACS-2 interacts with Bim and Bax to induce lysosomal membrane permeabilization, a step required for efficient TRAIL-mediated apoptosis in liver cells [17], [19], [44]. In the current study, simultaneous depletion of cIAP-1 and cIAP-2 results in PACS-2 accumulation and enhances PACS-2 translocation to the lysosomes following TRAIL-treatment. Functionally, this was accompanied by more efficient LMP, as demonstrated by the release of lysosomal cathepsins into the cytosol, and increased cell death. Further evidence that PACS-2 acts at the level of the lysosomes comes from the observation that PACS-2 deficient HuH-7 cells display both reduced LMP and sensitivity to TRAIL-induced apoptosis. More importantly, TRAIL sensitivity was not restored by a SMAC mimetic in PACS-2 deficient cells, confirming that IAPs act upstream of PACS-2-mediated LMP. Primary mouse hepatocytes are resistant to apoptosis induced by the recombinant TRAIL used in this study. Indeed, no apoptosis was detected in wild-type mouse hepatocytes after TRAIL-treatment; however, these cells were sensitized to apoptosis by the use of the SMAC mimetic, suggesting that IAPs may be involved in the intrinsic resistance of primary hepatocytes to TRAIL. On the contrary, inhibition of IAPs by the SMAC mimetic did not restore sensitivity to TRAIL-induced apoptosis in *Pacs-2^−/−^* mouse hepatocytes, in agreement with what observed in HuH-7 cells. TRAIL-induced apoptosis in *cIap1^−/−^* hepatocytes was not enhanced by the presence of the SMAC mimetic, but it was restored in *cIap2^−/−^* hepatocytes. This is consistent with the recent observation that in the absence of cIAP-1, cIAP-2 is unable to auto-ubiquitinate in response to SMAC mimetics, therefore it does not undergo proteasomal degradation and accumulates overtime [43]. The persistent high levels of cIAP-2 in *cIap1^−/−^* mouse hepatocytes likely account for their failure to undergo TRAIL-induced apoptosis even in the presence of a SMAC mimetic.

In conclusion, our results have identified PACS-2 as a substrate for the E3 ubiquitin ligase activity of cIAPs and unveiled a novel mechanism through which cIAPs modulate TRAIL-induced apoptosis in hepatobiliary cells. Loss of cIAPs reduces PACS-2 ubiquitination and results in PACS-2 accumulation, facilitating its translocation to the lysosomes and promoting TRAIL-induced cell death. These findings highlight the crucial role for cIAPs in regulating TRAIL-resistance, and demonstrate that cIAPs and PACS-2 antagonistically regulate TRAIL-induced LMP and apoptosis.
